# Internet-Based Interventions for Preventing Premature Birth in Preconceptional Women of Childbearing Age: Systematic Review

**DOI:** 10.2196/60690

**Published:** 2025-06-03

**Authors:** Sun-Hee Kim, Sun-Young Jung, Jin-Hwa Park, Jennie C De Gagne

**Affiliations:** 1 College of Nursing Research Institute of Nursing Science Daegu Catholic University Daegu Republic of Korea; 2 School of Nursing Duke University Durham, NC United States

**Keywords:** contraception, health promotion, high-risk behavior, internet-based interventions, preconception, premature birth, reproductive health, self-efficacy, systematic review

## Abstract

**Background:**

Preconception health is essential for preventing premature birth, yet engagement in preconception care remains low. Internet-based interventions offer scalable solutions, but their effectiveness in this context is underexplored.

**Objective:**

This systematic review aimed to describe the characteristics and designs of studies on internet-based interventions and evaluate their effectiveness in preventing premature birth among preconceptional women of childbearing age.

**Methods:**

We searched MEDLINE, Embase, CINAHL, and the Cochrane Library for randomized controlled trials and quasi-experimental studies on internet-based interventions targeting premature birth prevention, covering publications up to December 2023 with no language or geographic restrictions. The search, conducted initially in February 2023 and updated in March 2024, followed PRISMA (Preferred Reporting Items for Systematic reviews and Meta-Analyses) guidelines and was registered with PROSPERO (International Prospective Register of Systematic Reviews; CRD42021277024). Two reviewers independently screened studies, extracted data, and assessed the risk of bias using the revised Cochrane Risk of Bias tool. Due to heterogeneity in populations, interventions, and outcomes, a narrative synthesis was performed instead of a meta-analysis.

**Results:**

From 3437 articles identified across 2 searches, 9 studies were included after excluding duplicates and ineligible papers. Studies, primarily from high-income countries (eg, 4/9, 44% from the United States), varied in design (4/9, 44% randomized controlled trials; 5/9, 56% quasi-experimental) and timing (5/9, 56% post 2020). The overall risk of bias was high in 6/9 (67%) studies, with only 1/9 (11%) rated low risk. Interventions, delivered via websites (4/9, 44%), conversational agents (3/9, 33%), or other platforms, significantly improved reproductive health knowledge in 3/9 (33%) studies but showed no consistent impact on self-efficacy (no effect in 2/3 [67%] studies assessing it). Behavioral outcomes, such as folic acid use and contraception initiation, were inconsistent across 5/9 (56%) studies, with significant effects in short-term (eg, 2/9, 22%) but not long-term interventions (eg, 2/4, 50% at 12 months). No studies directly measured premature birth as an outcome.

**Conclusions:**

Internet-based interventions showed mixed effectiveness across reproductive health outcomes pertinent to premature birth prevention, with notable gains in knowledge but limited success in altering behaviors. Given the small number of studies and the prevalent high risk of bias, these findings warrant cautious interpretation. Future research, including robust clinical trials, is essential to develop, evaluate, and disseminate effective and safe internet-based interventions for preconception care.

**Trial Registration:**

PROSPERO CRD42021277024; https://www.crd.york.ac.uk/PROSPERO/view/CRD42021277024

## Introduction

### Background

Premature birth, defined as delivery before 37 weeks of gestation, is a major global public health concern [1]. It poses significant health risks for both mothers and infants, including long-term health complications and increased health care costs [2]. Despite advancements in prenatal care, premature birth rates remain high, and socioeconomic disparities exacerbate these risks [3]. Preconceptional health is pivotal in determining pregnancy outcomes, underscoring the importance of targeted interventions before conception [4].

Historically, efforts to reduce premature birth rates have primarily focused on enhancing prenatal care and addressing modifiable risk factors [5]. Preconception care, however, supports not only reproductive planning but also interventions aimed at minimizing risks, enabling women to start their pregnancies in optimal health and improving the chances of delivering a healthy newborn [6]. Extensive research indicates that addressing preconception risks can significantly improve maternal health and help prevent premature births [7,8]. However, traditional approaches often struggle to engage preconceptional women of childbearing age, limiting their effectiveness [9].

Advancements in digital technology have made internet-based interventions a promising, accessible, and cost-effective approach, delivering tailored, evidence-based support [10]. These interventions allow preconceptional women of childbearing age to engage flexibly, overcoming geographic and time barriers, while interactive features and real-time feedback address their diverse needs, promoting informed health decisions and behavior change [10]. Prior studies highlight the preconception period as an optimal time for mitigating unhealthy lifestyle behaviors, thereby enhancing health knowledge [11-13], promoting behavioral changes such as reducing risk factors like alcohol and smoking, increasing folate intake, and encouraging physical activity [11,12]. These interventions also focus on enhancing contraceptive use [13], updating vaccinations [12], and ultimately improving pregnancy outcomes [6,11,12]. However, the evidence from these reviews is generally weak due to the low quality of the literature, necessitating caution in interpretation [11-13]. Moreover, systematic reviews specifically examining internet-based interventions for preconceptional women of childbearing age, especially for preventing preterm births, are limited. Post COVID-19, there has been an uptick in the development of such interventions in various countries including the United States [14,15], France [16], Australia [17], and the Republic of Korea [18]. Therefore, this systematic review seeks to bridge this knowledge gap by synthesizing available evidence and assessing the impact of internet-based interventions on preventing preterm birth in preconceptional women of childbearing age.

### Objectives

We conducted a systematic review of randomized controlled trials (RCTs) and quasi-experimental studies focusing on internet-based interventions aimed at preventing premature births among preconceptional women of childbearing age. The objectives of this review were to (1) describe the general characteristics of the studies included, (2) identify the study designs used in internet-based interventions pertinent to premature birth prevention, and (3) evaluate the effectiveness of internet-based interventions in achieving outcomes related to premature birth prevention among the target population.

## Methods

### Design

This systematic review was reported in accordance with the PRISMA (Preferred Reporting Items for Systematic Reviews and Meta-Analyses) guidelines [19] (checklist in Multimedia Appendix 3) and was registered in PROSPERO (International Prospective Register of Systematic Reviews; CRD42021277024). Our review focused on 2 specific research questions formulated using the Population, Intervention, Comparison, and Outcome (PICO) framework: (1) What is the efficacy of internet-based interventions in reducing the risk of premature birth among preconceptional women of childbearing age compared to standard care? (2) How do internet-based interventions affect women’s health promotion and health outcomes to decrease the risk of premature birth among the target population? These questions were designed to assess the effectiveness of internet-based interventions in improving critical maternal health outcomes associated with the prevention of premature births. Our comprehensive search strategy targeted key electronic databases, including MEDLINE, Embase, CINAHL, and the Cochrane Library. The search strategy was developed with consultation from a researcher at the National Evidence-based Healthcare Collaborating Agency (NECA) in Korea, with 10 years of experience in systematic reviews and meta-analyses in health care technology and nursing, who performed the initial database searches. Studies were initially searched on February 28, 2023, with an update conducted in March 2024 covering publications up to December 2023. Additionally, we manually reviewed the study lists of all included publications to ensure a thorough investigation of relevant literature.

### Eligibility Criteria

Our inclusion criteria encompassed preconceptional women of childbearing age and interventions targeting premature birth prevention. Studies included randomized controlled trials (RCTs), quasi-experimental designs, and experimental studies with comparators focused on preventing premature birth via internet-based interventions requiring an internet connection. We imposed no restrictions regarding the country or language of publication. The target population included all women before pregnancy. Only internet-based interventions using various digital platforms, including computers and mobile phones, were included. There were no exclusion criteria for this review.

### Search Strategy

The search terms adapted for each database included a combination of terms related to population (eg, “women”), preconception (eg, “premature birth” and “prepregnancy”), information and communication technology (eg, “computer”), treatment (eg, “internet” and “online”), and study design (eg, “randomized controlled trial”). These terms were used to search titles, abstracts, keywords, or text words. The exact search terms are detailed in Multimedia Appendix 1.

### Selection and Data Collection Processes

The initial step involved importing all identified studies into a reference manager to eliminate duplicates. Subsequently, 2 reviewers (SHK and JHP) independently screened the titles and abstracts. Authors, fluent in English and Korean, translated non-English titles using a translation tool and reviewed English abstracts. Following this preliminary screening, relevant studies underwent a comprehensive full-text review. Any disagreements during this phase were resolved through discussion or consultation with a third reviewer (SYJ) to ensure consensus on study inclusion. Studies deemed irrelevant after the full-text review were excluded from further consideration. Concurrently, the reviewers collaboratively developed and pretested a data extraction form to systematically gather review characteristics and outcome data from the selected studies. The data extraction process was also independently conducted by 2 reviewers (SHK and JHP). In instances of discrepancies in the extracted data, the reviewers engaged in discussions to reach a consensus or consulted the third reviewers (SYJ and JCDG) for an objective resolution. A semi-automated method using EndNote (Clarivate) search and rating functions identified ineligible studies via keywords (eg, protocol, meta-analysis, and review), which were then manually excluded.

### Data Extraction

The extracted data comprised study characteristics (eg, authors, year, country of origin, research design, and sample size), study results (eg, primary and secondary findings for outcome measures, including effect sizes), and intervention details (eg, name, method, duration, and group type). Due to the variation in methodologies across studies, conducting a meta-analysis was deemed inappropriate. Instead, information was synthesized narratively, categorizing outcomes into reproductive health perception, reproductive health behaviors, and reproductive health status. Effect sizes were calculated using means and SDs or frequencies and percentages depending on the study design.

### Risk-of-Bias Assessment

A total of 2 reviewers independently evaluated the methodological quality using the revised Cochrane Risk-of-Bias (RoB) tool for randomized trials [20]. This tool assesses 5 domains: randomization process, deviation from intended interventions, missing outcome data, outcome measurement, and reported result selection grouped into 3 levels of RoB (low risk, some concern, and high risk). Studies were classified into 2 groups: intention-to-treat (ITT) and per-protocol (PP), with any disagreements resolved through discussion or consultation among the reviewers or with a third party.

### Statistical Analysis

Owing to the heterogeneity in interventions and participant characteristics, we opted for a narrative synthesis rather than a meta-analysis. When available, effect sizes were calculated using data from the studies, using various metrics such as Cohen *f*, Cohen *h*, odds ratio (*P* value and 95% CI), rate ratio (*P* value and 95% CI), and relative risk (*P* value and 95% CI) [21]. Of the 9 [22-30] papers reviewed, 1 (11%) [23] did not provide sufficient statistical data to calculate effect sizes for some variables. We attempted to contact the author of this study to obtain additional information about its results, but despite multiple inquiries, we received no response, preventing effect size calculations for that study. Consequently, effect sizes were calculated for 8 studies [22,24-30]. In cases where additional data from the original authors were not acquired, our evaluations relied solely on the information provided within the study itself.

## Results

### Overview

**Figure 1:**
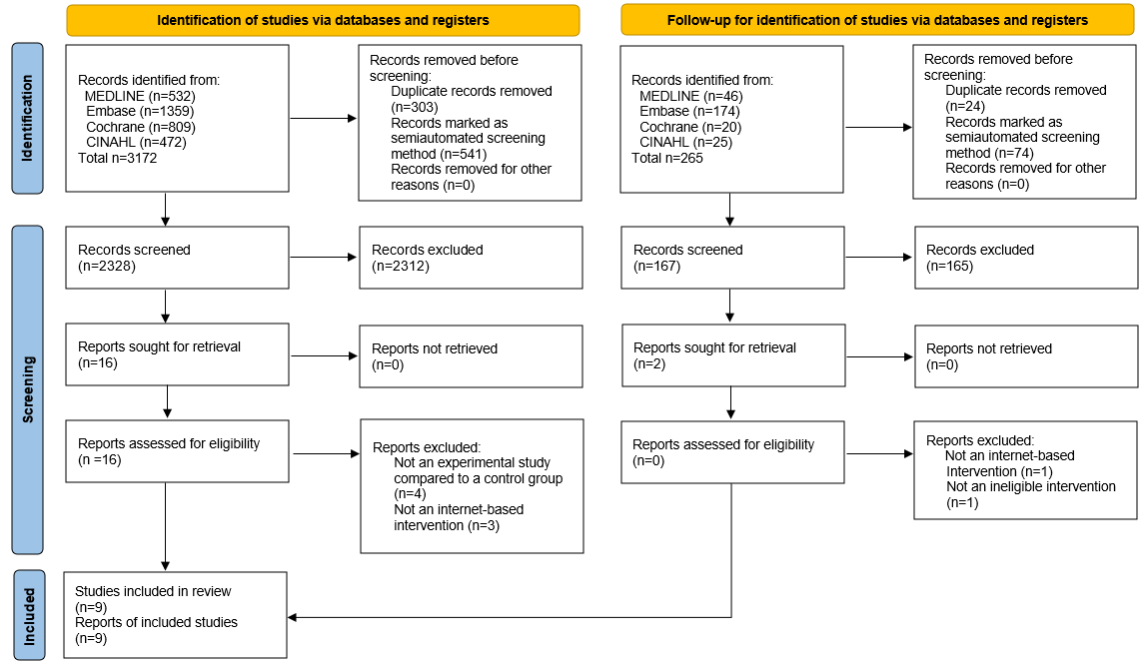
PRISMA (Preferred Reporting Items for Systematic Reviews and Meta-Analyses) diagram showing the selection of included studies.

Initially, a total of 3172 papers were retrieved from the four databases in February 2023. After removing 303 duplicates and 541 ineligible articles, 2328 remained. During the initial screening stage, 2312 papers were excluded after reviewing titles and abstracts. The full texts of the remaining 16 studies were then reviewed, and 7 were excluded: 4 lacked controlled comparators (one group pre- and postintervention, one group cohort, agreement test between data collection methods, and associative factors study), and 3 non-internet-based (1 teleconference and 2 computer-based). A subsequent search in March 2024 retrieved 265 studies from the same databases. After excluding 24 duplicates and 74 ineligible articles, 167 remained. During screening, 165 papers were excluded after reviewing the titles and abstracts. The full texts of the remaining 2 studies were reviewed and excluded: one used a live TV channel, and the other was an app predicting pregnancy likelihood, both ineligible as non–internet-based interventions. No additional studies were identified through a manual review of the included studies’ reference lists. Ultimately, 9 studies were selected for the systematic review, totaling 3437 articles screened across both searches after exclusions. Figure 1 illustrates the study selection process, updated to reflect these steps.

### Quality of the Studies and RoB Assessment

**Figure 2:**
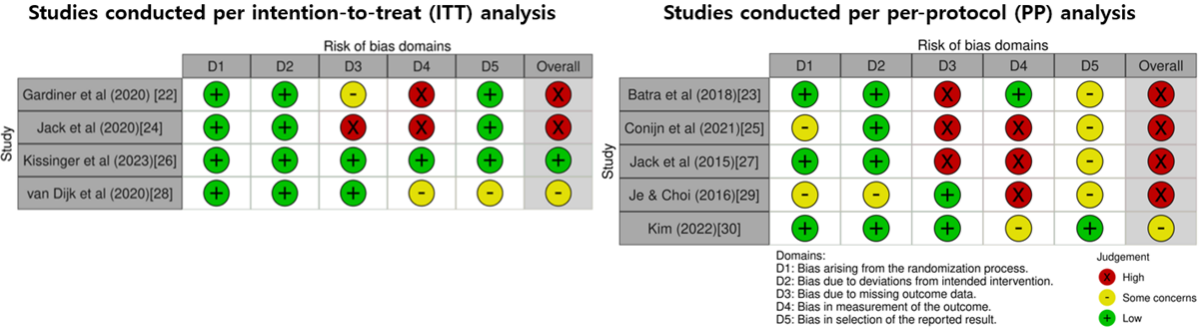
Risk-of-bias assessment using the revised Cochrane Risk-of-Bias tool for randomized trials.

In the subset of studies that used ITT analysis, the overall RoB was classified as low in 25% (1/4) of the studies. There were some concerns regarding bias in 25% (1/4) of the studies, and a high RoB was found in 50% (2/4) of the studies. Among the studies that used PP analysis, none (0/5) were assessed as having a low RoB; only 20% (1/5) of the studies had some concerns, while a high RoB was identified in 80% (4/5) of the studies. The detailed outcomes of the RoB assessment for the 4 ITT and 5 PP studies are illustrated in Figure 2 [22-30].

### Study Characteristics

The 9 unique studies in this review were conducted in the United States, the Netherlands (2/9, 22% studies [25,28]), and South Korea (2/9, 22% studies [29,30]), with most from the United States(5/9, 55% studies [22-24,26,27]). Publication years ranged from 2010 to 2022, with the majority (6/9, 66% studies) between 2020 and 2022. Most (7/9, 77%) studies used RCT designs, while some (2/9, 22% studies) used quasi-experimental approaches; 11% (1/9) [22] were secondary analyses, and the rest were primary studies. Theoretical frameworks varied, including the Transtheoretical Model and Health Belief Model, with one study unspecified [30]. Participants included nonpregnant women, couples with prepregnant or early-pregnant female partners, and Black teenage women. Detailed characteristics are in Tables 1 and 2.

**Table 1:** Overview of the general characteristics of the studies (N=9).

Characteristics, category, and studies		Values, n (%)
**Country**		
	The Netherlands [25,28]	2 (22)
	South Korea [29,30]	2 (22)
	United States [22-24,26,27]	5 (55)
**Publication year**		
	2010 -<2020 [23,27,29]	3 (33)
	≥2020 [22,24-26,28,30]	6 (66)
**Publication language**		
	English [22-28,30]	8 (88)
	Korean [29]	1 (11)
**Research design**		
	RCT^a^ [22-24,26-28,30]	7 (77)
	Quasi-experimental trial [25,29]	2 (22)
**Primary and secondary analysis**		
	Primary analysis study [23-30]	8 (88)
	Secondary analysis study [22]	1 (11)
**Theoretical framework**		
	Theory of Reasoned Action [23]	1 (11)
	Transtheoretical Model of Health Behavior Change [22,24,27]	3 (33)
	Behavior Change Model [28]	1 (11)
	Social Cognitive Theory combined with Gender and Power Theory [26]	1 (11)
	Health Belief Model [25,29]	2 (22)
	Not reported [30]	1 (11)
**Participants’ characteristics**		
	Nonpregnant women [22-24,27]	4 (44)
	Prepregnant couples [25,28,29]	3 (33)
	Women of childbearing age [30]	1 (11)
	Black teenaged women [26]	1 (11)
**Intervention type**		
	Website [23,26,29,30]	4 (44)
	Conversational agent-based [22,24,27]	3 (33)
	Mobile app [28]	1 (11)
	Video sharing platform [25]	1 (11)
**Intervention delivery method**		
	Individual [22-24,26,27,30]	6 (66)
	Couple [25,28,29]	3 (33)
**Intervention duration^d^**		
	≤1 week [30]	1 (11)
	1 week to ≤1 month [23,26,29]	3 (33)
	3 months to ≤6 months [27,28]	2 (22)
	6 months to ≤12 months [22,24]	2 (22)
	Not reported [25]	1 (11)
**Comparator intervention**		
	Suggestion a meeting with a health care provider [22,24,27]	3 (33)
	Standard health information [23,25,28]	3 (33)
	Attention control intervention [26]	1 (11)
	None [29,30]	2 (22)

^a^RCT: randomized controlled trial.

**Table 2:** Summary of the study designs for internet-based interventions on women of childbearing age (N=9).

Study, year	Country	Study design (analysis sets)	Participants and females’ age (intervention n/control n)	Experimental intervention	Intervention method and group type (I^a^ or C^b^)	Intervention duration	Comparative intervention
Batra et al [23], 2018	United States	2-armed RCT^c^ (ITT^d^)	Nonpregnant women, 18-45 years old (146/146)	MyFamilyPlan	Website (I)	2 weeks	Standard health information
Conijn et al [25], 2021	The Netherlands	2-armed quasi-experimental trial (PP^e^)	Prepregnant couples, 18-45 years old (789/781)	Educational online video about an autosomal recessive disorder	YouTube (C)	NR^f^	Standard health information
Gardiner et al [22], 2020	United States	2-armed RCT (ITT) Second analysis of Jack et al [24], 2020	Nonpregnant women, 18-34 years old from the previous study (240/240)	Gabby	Telephone and an embodied conversational agent system (I)	12 months	Suggestion a meeting with a health care provider
Jack et al [27], 2015	United States	2-armed RCT (PP)	Nonpregnant women, 18-34 years old (36/41)	Gabby	Telephone and an embodied conversational agent system (I)	6 months	Suggestion a meeting with a health care provider
Jack et al [24], 2020	United States	2-armed quasi-experimental trial (ITT)	Nonpregnant women, 18-34 years old (262/266)	Gabby	Telephone and an embodied conversational agent system (I)	12 months	Suggestion a meeting with a health care provider
Je and Choi, 2016 [29]	South Korea	2-armed quasi-experimental trial (PP)	Premarital couples, 20-39 years old (26/25)	Preconception Health Promotion	Website (C)	4 weeks	None
Kim, 2022 [30]	South Korea	2-armed RCT (PP)	Women of childbearing age, 19-49 years old (49/49)	Webtoon education program on preventive self-management related to premature labor	Website (I)	2 days	None
Kissinger et al, 2023 [26]	United States	2-armed RCT (ITT and PP)	Black teenage women, 18-19 years old (315/322)	Be yoU, Talented, Informed, Fearless, Uncompromised, and Loved	Website (I)	4 weeks	Attention control intervention
Van Dijk et al, 2020 [28]	The Netherlands	2-armed RCT (ITT)	Women contemplating pregnancy or <13 weeks pregnant and their male partners, 18-45 years old (109/109)	Smarter Pregnancy	Mobile application (C)	24 weeks	Standard health information

^a^I: individual.

^b^C: couple.

^c^RCT: randomized controlled trial.

^d^ITT: intention-to-treat.

^e^PP: per-protocol.

^f^NR: not reported.

### Intervention Characteristics

#### Intervention Method and Group Type

The 9 studies delivered interventions mainly via websites (4/9, 44% studies [23,26,29,30]) and conversational agents (3/9, 33% studies [22,24,27]), with one using a mobile app (1/9, 11% study [28]) and another a video-sharing platform (YouTube; 1/9, 11% study [25]). Most targeted individual participants, while 33% (3/9) of the studies focused on couples [25,28,29]. Further details are in Tables 1 and 2.

#### Intervention Duration, and Comparative Approaches

Intervention durations ranged from one week or less [30] to 12 months [22,24], with one unspecified [25]. Comparative interventions included standard health information (3/9, 33% studies [23,25,28]), meetings with health care providers (3/9, 33% studies [22,24,27]), an attention control (1/9, 11% study [26]), or no intervention (2/9, 22% studies [29,30]). For further details, see Tables 1 and 2.

#### Outcomes and Effects of Interventions

The outcomes of the 9 interventions, detailed in Multimedia Appendix 2 [22-30], were categorized into reproductive health perception and behaviors, each with 4 subcategories linked to premature birth prevention (Table 3).

**Table 3:** Categories of outcome measures of internet-based intervention on women of childbearing age.

Categories and subcategories		Outcomes measurements
**Reproductive health perception**		
	Self-efficacy of reproductive health	Self-efficacy on preconception health attitude and behavior [23] Preventive health management self-efficacy related to premature labor [28] Perceived self-efficacy of preconception health promotion [29]
	Perception of reproductive health promotion	Self-perception of awareness of preconception health promotion [29] Content awareness of preconception health promotion [29] Perceived benefits of preconception health promotion [29] Perceived barriers to preconception health promotion [29]
	Knowledge of reproductive health	Preventive self-management knowledge related to premature labor [30] Genetic knowledge [25]
	Perception of hereditary disorders	Perceived severity of mucopolysaccharidosis III [25]: Mucopolysaccharidosis III is a severe disease Mucopolysaccharidosis III has a very bad life expectancy Perceived risk of hereditary disorder [25] Being a carrier of a severe hereditary disease as (very) high risk Both partners are carriers of the same disease as (very) high risk Having a child with a severe hereditary disorder as (very) high risk
**Reproductive health behaviors**		
	Self-management for reproductive health	Self-reported discussion of reproductive health with provider [23] Scheduling an additional appointment to address her reproductive health after her well-woman visit [23] Reproductive health promotion behavior [29]
	Dietary and nutritional intake	Initiating folate supplementation [23] Folic acid supplement use [28] Dietary risk score [28] Vegetable intake [28] Fruit intake [28]
	Behavioral promotion on preconception care risks	Preconception care risks from the nutrition domain [22] At 6 months, risks that progressed At 6 months, risks at action or maintenance At 12 months, risks that progressed At 12 months, risks at action or maintenance Reductions in proportion of preconception care risks [27] Reductions in number of preconception care risks per person [27] Preconception care risks [24] At 6 months, risks at action or maintenance At 12 months, risks at action or maintenance At 6 months, risks that progressed forward At 6 months, risks that regressed backward At 12 months, risks that progressed forward At 12 months, risks that regressed backward
	Contraception use	Initiating or changing the birth control method [23] Initiating reliable contraception use [26] Intention to use reliable contraception in the next year [26] Initiating dual methods of contraception [26] Intention to use condoms [26]

#### Reproductive Health Perception

This domain included outcomes like self-efficacy, perception of health promotion, knowledge, and hereditary disorder perception. Studies assessed self-efficacy in areas such as preconception health attitudes [23], health promotion [29], and preventive management for premature labor [30], with significant gains only in the latter using web cartoons [30]. Perception of health promotion showed improvements in awareness and benefits in one study [29]. Knowledge of reproductive health, including genetic awareness [25] and premature labor management [30], increased significantly in 22% studies (2/9). Perception of hereditary disorders, explored in one(11%) study [25], improved for severity and risk, though not for carrier risk perception.

#### Reproductive Health Behaviors

This area covered self-management, nutrition, preconception care risk reduction, and contraception use. Self-management studies showed mixed results, with gains in provider discussions [23] and promotion behaviors [29], but not appointment scheduling [23]. Nutrition interventions enhanced dietary scores and vegetable intake [23,28], though folic acid and fruit intake remained unchanged [28]. Preconception care risk reduction efforts reduced risks significantly in some studies [22,24,27]; for example, at 6 months, the Gabby group resolved 27.8% of preconception care risks (8.3/23.2) versus 20.5% (5.5/24.2) in the control group [27]. The Gabby system significantly reduced risks among African American women, with behavior change rising over 6 months and sustained at 12 months, although no further progress occurred beyond 12 months [24]. Contraception use improved in initiating methods [23] and intent for reliable use or condoms [26], although immediate effects varied. See Table 3 for detailed outcomes.

## Discussion

### Overview

Internet-based interventions showed mixed effectiveness in improving reproductive health outcomes for premature birth prevention among preconceptional women of childbearing age, with notable knowledge gains but inconsistent behavioral impacts.

### Principal Results

This systematic review provides a comprehensive evaluation of internet-based interventions aimed at preventing premature birth in preconceptional women of childbearing age. Only 9 studies directly addressed this topic, revealing a significant gap in online preconception health interventions. This scarcity aligns with prior reviews noting limited digital strategies in preconception care [31,32], despite the reported potential of mobile apps to enhance behaviors like weight management [33], physical activity, and nutrition [33,34]. Although these technologies offer cost-effective, accessible care in other domains [35], apps tailored for preconception health lag behind broader health tech development. Post-COVID-19, reliance on digital health solutions has grown [36,37], yet this surge has not extended to preconception health, highlighting a key area for future innovation [38]. Prioritizing mobile apps that leverage smartphone accessibility could reduce premature birth risks and improve reproductive health.

The reviewed studies focused primarily on health promotion, with one addressing genetic history, leaving most of the 14 preconception care domains (eg, immunization and medical conditions) unexplored [39]. We anticipated more interventions targeting premature birth prevention directly, but the 9 studies often emphasized broader health outcomes (e.g., disease prevention) with indirect links to this goal, contributing to the limited scope [40]. This gap mirrors challenges in wider reproductive health interventions, underscoring the need for comprehensive digital strategies.

### Comparison With Prior Work

The majority of the studies we reviewed were conducted in high-income countries such as the United States, the Netherlands, and South Korea [41], where the widespread availability of the internet and digital devices facilitates the implementation of web-based interventions. An analysis of the publication years within this study reveals a growing trend in these interventions, with more than half of the studies conducted after 2020. The COVID-19 pandemic acted as a catalyst for rapid advancements in mobile technology, significantly enhancing the capability of individuals to connect with healthcare systems and access essential health-related guidance [42]. This period also witnessed a notable surge in the development of health and fitness apps, with the iOS app market seeing an unexpected growth of 29.9% in the availability of these apps postpandemic [38]. However, the development of apps specifically for preconception health has not kept pace with these technological advancements, indicating a significant opportunity for innovation and enhancement in this specific area of health technology.

In terms of intervention duration, our review found that the length of internet-based interventions was strategically aligned with their cognitive and behavioral targets. Short-term interventions, lasting less than one month, typically focused on assessing the effects on health-related beliefs [25,29], knowledge [25,30], intention [26], and self-efficacy [23,30]. In contrast, longer interventions, those exceeding 80 days, aimed to initiate or modify behaviors [22,24,27,28]. The efficacy of these long-term interventions in fostering healthful behaviors, curbing unhealthy ones, maintaining behavioral changes in physical activity, and promoting sustained abstinence from substance abuse highlights their critical role [43]. As a result, trials designed to enhance health awareness or beliefs often opted for shorter durations, while those seeking substantive behavioral change generally favored longer-term interventions to achieve more profound and lasting impacts.

The reliability of the studies reviewed was predominantly questionable, with only one out of the nine studies (11%) exhibiting a low RoB [22]. The majority displayed moderate to high risk, attributed to significant issues like extensive missing outcome data, the reliance on self-reported outcome measures, and the absence of blinding regarding intervention status. These issues highlight the critical need for more rigorously designed high-quality studies within this domain. Internet-based interventions pose inherent challenges such as the difficulty of blinding and participant attrition, which can influence the accuracy of self-reported data. Therefore, RCTs must be meticulously planned to mitigate these biases, taking into consideration the specific characteristics of participants during data analysis and interpretation.

In terms of intervention effectiveness, internet-based approaches targeting enhancements in reproductive health perception—specifically focusing on reproductive health promotion, knowledge, and the perception of hereditary disorders—were predominantly short-term and utilized platforms such as websites or YouTube. While statistically significant effects were observed in these areas [23,25,29,30], except for the perceived risk of hereditary disorders [25], caution is warranted. The robustness of these findings is questionable as the results for the perception of reproductive health promotion and hereditary disorders are each derived from a single study, and the insights on knowledge of reproductive health from just two studies.

Furthermore, there were no consistent statistically significant effects noted in self-efficacy related to preconception health attitudes and behaviors [23], nor in the perceived self-efficacy of preconception health promotion [29]. This is in contrast to a non-internet-based systematic literature review [40], which reported significant improvements in participants' knowledge and self-efficacy concerning reproductive life planning, albeit these findings were considered of low quality. Such disparities underscore the urgent need for additional high-quality research to develop and refine internet-based interventions that effectively enhance reproductive health perception. Future studies should focus on improving intervention strategies, extending durations, and refining methods to support evidence-based enhancements in preconception health behaviors.

In this study, specific subcategories of reproductive health behaviors were examined, with only two studies assessing each of self-management for reproductive health [23,29] and dietary and nutritional intake [23,28]. The limited number of studies, coupled with inconsistencies in measurement approaches, has resulted in insufficient evidence to conclusively determine their effectiveness. Additionally, subcategories focusing on behavioral promotion to mitigate preconception care risks were evaluated through long-term interventions. While the results demonstrated effectiveness at the 6-month mark across all relevant studies [22,24,27], the assessments at the 12-month mark indicated only partial effectiveness [22,24]. This variability highlights the crucial need for more research to validate and refine these interventions. Furthermore, the analysis of contraception uses across two studies [23,26] revealed inconsistencies in the effectiveness of initiating birth control methods, underscoring the challenges in achieving consistent outcomes across diverse demographic and temporal contexts. For instance, the “MyFamilyPlan” program targeted non-pregnant women over a brief two-week period with immediate postintervention evaluation [23], whereas the “Be yoU, Talented, Informed, Fearless, Uncompromised, and Loved (BUtiful)” program engaged Black teenage women over four weeks, with assessments extending to 6 and 12 months [26]. These differences exemplify the complex dynamics in implementing and evaluating internet-based interventions, which can significantly affect outcomes. These disparities necessitate a cautious approach to interpreting results and call for a more nuanced understanding of how different factors influence the effectiveness of contraception interventions. Additionally, broader reviews in reproductive health interventions [12] have also pointed to a scarcity of robust data, with only a few studies examining outcomes like folic acid supplementation, vaccination uptake, increased physical activity, and smoking and alcohol consumption reduction. These studies were generally rated as having weak to moderate quality, further emphasizing the ongoing need for high-quality research that can establish effective, reliable interventions in these critical areas of reproductive health.

### Strengths and Limitations

This review offers a broad examination of internet-based interventions for premature birth prevention, with inclusive criteria enhancing global relevance. However, limitations persist. First, restricting searches to four databases may have missed relevant studies; broader database inclusion could improve coverage. Second, focusing on experimental designs excluded qualitative insights, potentially limiting contextual depth; mixed methods could enrich future reviews. Third, omitting grey literature (e.g., dissertations) risked overlooking emerging findings, especially in digital health; including such sources might mitigate this, though publication bias was addressed via rigorous search strategies. Fourth, reliance on high-internet regions may skew generalizability, affecting applicability in low-access areas—future studies should diversify settings. Finally, high RoB in most studies, due to bias from self-reports and attrition, weakened conclusions; this reflects primary research quality and calls for robust trials.

### Conclusions

This systematic review critically evaluates the efficacy of internet-based interventions in improving preconception health and preventing premature birth among women of childbearing age. The findings reveal that while some interventions successfully enhanced knowledge of reproductive health, they were less effective in altering health behaviors such as contraception use and dietary supplement intake. The inconsistent results across different domains suggest a complex interaction between intervention design and participant engagement, underscoring the challenges of implementing digital health strategies effectively. This variability, coupled with the high risk of bias in many studies, highlights critical areas for improvement in research methodology and intervention design. Moving forward, there is a clear need for more rigorous, well-designed studies that not only refine these interventions but also expand their scope to more comprehensively address the multifaceted needs of preconception care. Such research should aim to harness the full potential of digital technology in public health to create more nuanced and impactful interventions that can significantly enhance outcomes for women globally.

## Appendix

Multimedia Appendix 1 Search terms on databases.

Multimedia Appendix 2 Outcome measures and effects of internet-based intervention on preconceptional women of childbearing age.

Multimedia Appendix 3 PRISMA Checklist.

## Abbreviations

ITT: intention-to-treat
NECA: National Evidence-based Healthcare Collaborating Agency
PICO: Population, Intervention, Comparison, and Outcome
PP: per-protocol
PRISMA: Preferred Reporting Items for Systematic Reviews and Meta-Analyses
PROSPERO: International Prospective Register of Systematic Reviews
RCT: randomized controlled trial
RoB: risk of bias
