# Supplementary material for: Internet-Based Interventions for Preventing Premature Birth in Preconceptional Women of Childbearing Age: Systematic Review
Source: J Med Internet Res. 2025 Jun 3;27:e60690. doi: 10.2196/60690 (PMC12174874; doi:10.2196/60690)
Supplement: Multimedia Appendix 1 [file jmir_v27i1e60690_app1.docx]

**Table S1.**

| **Search date** | **February 28, 2023** | | |
| --- | --- | --- | --- |
| **Databases** | **No** | **Search terms** | **Results** |
| Ovid-MEDLINE  (1946 - Feb 28, 2023) | 1 | Wom?n.tw. OR female.tw. OR women/ | 2,069,744 |
|  | 2 | prematur$.tw. OR preterm.tw. OR [Premature Birth](https://ovidsp.dc2.ovid.com/ovid-a/ovidweb.cgi?&Controlled+Vocabulary=Mapping%7c4&Return=mapping&S=PJFAFPGIFHEBEBCOJPPJPEHGHKGDAA00)/ OR [Obstetric Labor, Premature](https://ovidsp.dc2.ovid.com/ovid-a/ovidweb.cgi?&Controlled+Vocabulary=Mapping%7c8&Return=mapping&S=PJFAFPGIFHEBEBCOJPPJPEHGHKGDAA00)/ OR PTB.tw. OR preconception.tw. OR prepregnancy.tw. OR (pregnanc$ adj1 (high-risk OR problem$ OR complicat$)).tw. OR Pregnancy, High-Risk/ OR Pregnancy Complications/ OR Preconception Care/ | 354,531 |
|  | 3 | (video OR $computer OR television OR virtual OR VR OR animation OR ((command-line OR graphic user) adj1 interface)).tw. OR Virtual Reality Exposure Therapy/ OR User-Computer Interface/ | 475,105 |
|  | 4 | ((distance OR online OR on-line OR electronic OR web-based OR internet OR correspondence) AND (education OR learning OR $schooling OR courses OR intervention OR program)).tw. OR Education, Distance/ OR Computer-Assisted Instruction/ OR Internet/ | 223,047 |
|  | 5 | ((randomized controlled trial OR controlled clinical trial).pt. OR randomized.ab. OR randomised.ab. OR placebo.ab. OR drug therapy.fs. OR randomly.ab. OR trial.ab. OR groups.ab. OR experimental.tw.) NOT (exp animals/ NOT humans.sh.) | 5,594,548 |
|  | 6 | 1 AND 2 AND (3 OR 4) AND 5 | 532 |
| Ovid-EMBASE  (1974 - Feb 28, 2023) | 1 | Wom?n.tw. OR female.tw. OR female/ | 11,645,442 |
|  | 2 | prematur$.tw. OR prematurity/ OR preterm.tw. OR [premature labor](https://ovidsp.dc2.ovid.com/ovid-a/ovidweb.cgi?&Controlled+Vocabulary=Mapping%7c4&Return=mapping&S=PJFAFPGIFHEBEBCOJPPJPEHGHKGDAA00)/ OR PTB.tw. OR preconception.tw. OR prepregnancy.tw. OR prepregnancy care/ OR (pregnanc$ adj1 (high-risk OR problem$ OR complicat$)).tw. OR high risk pregnancy/ OR pregnancy complication/ | 456,193 |
|  | 3 | (video OR $computer OR television OR virtual OR VR OR animation OR ((command-line OR graphic user) adj1 interface)).tw. OR virtual reality exposure therapy/ OR virtual reality/ OR computer interface/ | 617,035 |
|  | 4 | ((distance OR online OR on-line OR electronic OR web-based OR internet OR correspondence) AND (education OR learning OR $schooling OR courses OR intervention OR program)).tw. OR e-learning/ OR computer interface/ OR Internet/ | 348,962 |
|  | 5 | (Randomized controlled trial/ OR Controlled clinical study/ OR random$.ti,ab. OR randomization/ OR intermethod comparison/ OR placebo.ti,ab. OR (compare OR compared OR comparison).ti. OR ((evaluated OR evaluate OR evaluating OR assessed OR assess) and (compare OR compared OR comparing OR comparison)).ab. OR (open adj label).ti,ab. OR ((double OR single OR doubly OR singly) adj (blind OR blinded OR blindly)).ti,ab. OR double blind procedure/ OR parallel group$1.ti,ab. OR (crossover OR cross over).ti,ab. OR ((assign$ OR match OR matched OR allocation) adj5 (alternate OR group$1 OR intervention$1 OR patient$1 OR subject$1 OR participant$1)).ti,ab. OR experimental.ti, ab. OR (assigned OR allocated).ti,ab. OR (controlled adj7 (study OR design OR trial)).ti,ab. OR (volunteer OR volunteers).ti,ab. OR human experiment/ OR trial.ti.) not (((random$ adj sampl$ adj7 ("cross section$" OR questionnaire$1 OR survey$ OR database$1)).ti,ab. not (comparative study/ OR controlled study/ OR randomi?ed controlled.ti,ab. OR randomly assigned.ti,ab.)) OR (Cross-sectional study/ not (randomized controlled trial/ OR controlled clinical study/ OR controlled study/ OR randomi?ed controlled.ti,ab. OR control group$1.ti,ab.)) OR (((case adj control$) and random$) not randomi?ed controlled).ti,ab. OR (Systematic review not (trial OR study)).ti. OR (nonrandom$ not random$).ti,ab. OR "Random field$".ti,ab. OR (random cluster adj3 sampl$).ti,ab. OR ((review.ab. and review.pt.) not trial.ti.) OR ("we searched".ab. and (review.ti. OR review.pt.)) OR "update review".ab. OR (databases adj4 searched).ab. OR ((rat OR rats OR mouse OR mice OR swine OR porcine OR murine OR sheep OR lambs OR pigs OR piglets OR rabbit OR rabbits OR cat OR cats OR dog OR dogs OR cattle OR bovine OR monkey OR monkeys OR trout OR marmoset$1).ti. and animal experiment/) OR (Animal experiment/ not (human experiment/ OR human/))) | 6,302,357 |
|  | 6 | 1 AND 2 AND (3 OR 4) AND 5 | 1,359 |
| Cochrane Central Register of Controlled Trials (1995 - Feb 28, 2023) | 1 | (Wom*n OR female):ti,ab,kw OR mh "Women" | 929,495 |
|  | 2 | (prematur* OR preterm OR PTB OR preconception OR prepregnancy OR (pregnanc*NEXT/1(high-risk OR problem$ OR complicat$))):ti,ab,kw OR mh "Premature Birth" OR mh "Pregnancy, High-Risk" OR mh "Obstetric Labor, Premature" OR mh "Pregnancy Complications" OR mh "Preconception Care" | 66,482 |
|  | 3 | (video OR $computer OR television OR virtual OR VR OR animation OR ((command-line OR graphic user) adj1 interface)).tw. OR mh "Video Games" OR mh "Virtual Reality Exposure Therapy" OR mh "Virtual Reality" OR mh "User-Computer Interface" | 10,536 |
|  | 4 | ((distance OR online OR on-line OR electronic OR web-based OR internet OR correspondence) AND (education OR learning OR $schooling OR courses OR intervention OR program)).tw. OR mh "Education, Distance" OR mh "Internet" | 10,739 |
|  | 5 | #3 OR #4 | 10,801 |
|  | 6 | "randomized controlled trial":pt OR "controlled clinical trial":pt OR "randomized":ti,ab,kw OR "randomly":ti,ab,kw OR "trial":ti,ab,kw OR "random":ti,ab,kw OR "RCT":ti,ab,kw OR "Randomization":ti,ab,kw OR "Quasi-Experimental":ti,ab,kw OR "Experimental Studies":ti,ab,kw | 1,351,095 |
|  | 7 | #1 AND #2 AND #5 AND #6 | 809 |
| EBSCO CINAHL (1993 - Feb 28, 2023) | 1 | Wom*n OR female | 17,252,863 |
|  | 2 | ( Premature* OR preterm OR prepregnancy ) OR ( Pregnanc* AND (high-risk OR problem* OR complicat*) ) | 1,248,324 |
|  | 3 | ( video OR *computer OR television OR virtual OR VR OR animation ) OR ( (command-line OR graphic user) AND interface ) OR ( ((distance OR online OR on-line OR electronic OR web-based OR internet OR correspondence) AND (education OR learning OR *schooling OR courses OR intervention OR program) ) | 12,181,930 |
|  | 4 | (AB("randomized controlled trial" OR "controlled clinical trial" OR "randomized" OR "randomly" OR "trial" OR "random" OR "RCT" OR "Randomization" OR "Quasi-Experimental")) OR "Experimental Studies" | 5,413,222 |
|  | 5 | 1 AND 2 AND 3 AND 4 | 534 |
| **Search date** | **March 14, 2024** | | |
| **Databases** | **No** | **Search terms** | **Results** |
| Ovid-MEDLINE  (Jan 01, 2023-Dec 31, 2023) | 1 | Wom?n.tw. OR female.tw. OR women/ | 2,196,905 |
|  | 2 | limit 1 to yr="2023" | 126,929 |
|  | 3 | prematur$.tw. OR preterm.tw. OR [Premature Birth](https://ovidsp.dc2.ovid.com/ovid-a/ovidweb.cgi?&Controlled+Vocabulary=Mapping%7c4&Return=mapping&S=PJFAFPGIFHEBEBCOJPPJPEHGHKGDAA00)/ OR [Obstetric Labor, Premature](https://ovidsp.dc2.ovid.com/ovid-a/ovidweb.cgi?&Controlled+Vocabulary=Mapping%7c8&Return=mapping&S=PJFAFPGIFHEBEBCOJPPJPEHGHKGDAA00)/ OR PTB.tw. OR preconception.tw. OR prepregnancy.tw. OR (pregnanc$ adj1 (high-risk OR problem$ OR complicat$)).tw. OR Pregnancy, High-Risk/ OR Pregnancy Complications/ OR Preconception Care/ | 370,800 |
|  | 4 | limit 3 to yr="2023" | 16,339 |
|  | 5 | (video OR $computer OR television OR virtual OR VR OR animation OR ((command-line OR graphic user) adj1 interface)).tw. OR Virtual Reality Exposure Therapy/ OR User-Computer Interface/ | 510,509 |
|  | 6 | limit 5 to yr="2023" | 35,765 |
|  | 7 | ((distance OR online OR on-line OR electronic OR web-based OR internet OR correspondence) AND (education OR learning OR $schooling OR courses OR intervention OR program)).tw. OR Education, Distance/ OR Computer-Assisted Instruction/ OR Internet/ | 247,069 |
|  | 8 | limit 7 to yr="2023" | 24,254 |
|  | 9 | ((randomized controlled trial OR controlled clinical trial).pt. OR randomized.ab. OR randomised.ab. OR placebo.ab. OR drug therapy.fs. OR randomly.ab. OR trial.ab. OR groups.ab. OR experimental.tw.) NOT (exp animals/ NOT humans.sh.) | 5,929,144 |
|  | 10 | limit 9 to yr="2023" | 330,099 |
|  | 11 | 2 AND 4 AND (6 OR 8) AND 10 | 46 |
| Ovid-EMBASE  (Jan 01, 2023-Dec 31, 2023) | 1 | Wom?n.tw. OR female.tw. OR female/ | 12,206,599 |
|  | 2 | limit 1 to yr="2023" | 721,294 |
|  | 3 | prematur$.tw. OR prematurity/ OR preterm.tw. OR [premature labor](https://ovidsp.dc2.ovid.com/ovid-a/ovidweb.cgi?&Controlled+Vocabulary=Mapping%7c4&Return=mapping&S=PJFAFPGIFHEBEBCOJPPJPEHGHKGDAA00)/ OR PTB.tw. OR preconception.tw. OR prepregnancy.tw. OR prepregnancy care/ OR (pregnanc$ adj1 (high-risk OR problem$ OR complicat$)).tw. OR high risk pregnancy/ OR pregnancy complication/ | 476,981 |
|  | 4 | limit 3 to yr="2023" | 25,603 |
|  | 5 | (video OR $computer OR television OR virtual OR VR OR animation OR ((command-line OR graphic user) adj1 interface)).tw. OR virtual reality exposure therapy/ OR virtual reality/ OR computer interface/ | 654,102 |
|  | 6 | limit 5 to yr="2023" | 43,864 |
|  | 7 | ((distance OR online OR on-line OR electronic OR web-based OR internet OR correspondence) AND (education OR learning OR $schooling OR courses OR intervention OR program)).tw. OR e-learning/ OR computer interface/ OR Internet/ | 377,866 |
|  | 8 | limit 7 to yr="2023" | 32,135 |
|  | 9 | (Randomized controlled trial/ OR Controlled clinical study/ OR random$.ti,ab. OR randomization/ OR intermethod comparison/ OR placebo.ti,ab. OR (compare OR compared OR comparison).ti. OR ((evaluated OR evaluate OR evaluating OR assessed OR assess) and (compare OR compared OR comparing OR comparison)).ab. OR (open adj label).ti,ab. OR ((double OR single OR doubly OR singly) adj (blind OR blinded OR blindly)).ti,ab. OR double blind procedure/ OR parallel group$1.ti,ab. OR (crossover OR cross over).ti,ab. OR ((assign$ OR match OR matched OR allocation) adj5 (alternate OR group$1 OR intervention$1 OR patient$1 OR subject$1 OR participant$1)).ti,ab. OR experimental.ti, ab. OR (assigned OR allocated).ti,ab. OR (controlled adj7 (study OR design OR trial)).ti,ab. OR (volunteer OR volunteers).ti,ab. OR human experiment/ OR trial.ti.) not (((random$ adj sampl$ adj7 ("cross section$" OR questionnaire$1 OR survey$ OR database$1)).ti,ab. not (comparative study/ OR controlled study/ OR randomi?ed controlled.ti,ab. OR randomly assigned.ti,ab.)) OR (Cross-sectional study/ not (randomized controlled trial/ OR controlled clinical study/ OR controlled study/ OR randomi?ed controlled.ti,ab. OR control group$1.ti,ab.)) OR (((case adj control$) and random$) not randomi?ed controlled).ti,ab. OR (Systematic review not (trial OR study)).ti. OR (nonrandom$ not random$).ti,ab. OR "Random field$".ti,ab. OR (random cluster adj3 sampl$).ti,ab. OR ((review.ab. and review.pt.) not trial.ti.) OR ("we searched".ab. and (review.ti. OR review.pt.)) OR "update review".ab. OR (databases adj4 searched).ab. OR ((rat OR rats OR mouse OR mice OR swine OR porcine OR murine OR sheep OR lambs OR pigs OR piglets OR rabbit OR rabbits OR cat OR cats OR dog OR dogs OR cattle OR bovine OR monkey OR monkeys OR trout OR marmoset$1).ti. and animal experiment/) OR (Animal experiment/ not (human experiment/ OR human/))) | 6,599,457 |
|  | 10 | limit 9 to yr="2023" | 378,256 |
|  | 11 | 2 AND 4 AND (6 OR 8) AND 10 | 174 |
| Cochrane Central Register of Controlled Trials (Jan 01, 2023-Dec 31, 2023) | 1 | (Wom*n OR female):ti,ab,kw OR mh "Women" | 58,210 |
|  | 2 | (prematur* OR preterm OR PTB OR preconception OR prepregnancy OR (pregnanc*NEXT/1(high-risk OR problem$ OR complicat$))):ti,ab,kw OR mh "Premature Birth" OR mh "Pregnancy, High-Risk" OR mh "Obstetric Labor, Premature" OR mh "Pregnancy Complications" OR mh "Preconception Care" | 4,636 |
|  | 3 | (video OR $computer OR television OR virtual OR VR OR animation OR ((command-line OR graphic user) adj1 interface)).tw. OR mh "Video Games" OR mh "Virtual Reality Exposure Therapy" OR mh "Virtual Reality" OR mh "User-Computer Interface" | 572 |
|  | 4 | ((distance OR online OR on-line OR electronic OR web-based OR internet OR correspondence) AND (education OR learning OR $schooling OR courses OR intervention OR program)).tw. OR mh "Education, Distance" OR mh "Internet" | 585 |
|  | 5 | #3 OR #4 | 594 |
|  | 6 | "randomized controlled trial":pt OR "controlled clinical trial":pt OR "randomized":ti,ab,kw OR "randomly":ti,ab,kw OR "trial":ti,ab,kw OR "random":ti,ab,kw OR "RCT":ti,ab,kw OR "Randomization":ti,ab,kw OR "Quasi-Experimental":ti,ab,kw OR "Experimental Studies":ti,ab,kw | 98,815 |
|  | 7 | #1 AND #2 AND #5 AND #6 | 20 |
| EBSCO CINAHL (Jan 01, 2023-Dec 31, 2023) | 1 | Wom*n OR female | 89,640 |
|  | 2 | ( Premature* OR preterm OR prepregnancy ) OR ( Pregnanc* AND (high-risk OR problem* OR complicat*) ) | 8,556 |
|  | 3 | ( video OR *computer OR television OR virtual OR VR OR animation ) OR ( (command-line OR graphic user) AND interface ) OR ( ((distance OR online OR on-line OR electronic OR web-based OR internet OR correspondence) AND (education OR learning OR *schooling OR courses OR intervention OR program) ) | 32,177 |
|  | 4 | (AB("randomized controlled trial" OR "controlled clinical trial" OR "randomized" OR "randomly" OR "trial" OR "random" OR "RCT" OR "Randomization" OR "Quasi-Experimental")) OR "Experimental Studies" | 20,830 |
|  | 5 | 1 AND 2 AND 3 AND 4 | 155 |
