# Supplementary material for: Internet-Based Interventions for Preventing Premature Birth in Preconceptional Women of Childbearing Age: Systematic Review
Source: J Med Internet Res. 2025 Jun 3;27:e60690. doi: 10.2196/60690 (PMC12174874; doi:10.2196/60690)
Supplement: Multimedia Appendix 2 [file jmir_v27i1e60690_app2.docx]

Multimedia Appendix 2. Outcome measures and effects of internet-based intervention on preconceptional women of childbearing age (N=9).

| Outcomes | Outcome measures [References] | Effect size (between groups comparison) | p-value (confidence interval) | Effect of intervention |
| --- | --- | --- | --- | --- |
| Reproductive health perception | | | |  |
| *Self-efficacy of reproductive health* | |  |  |  |
|  | Self-efficacy on preconception health attitude and behavior [23] | NR^a^ | NR | No effect |
|  | Preventive health management self-efficacy related to premature labor [30] | f^b^=0.161 | Changes in T0–T1, in T0–T2:  .002 (0.02, 0.11), <.001 (0.12, 0.30) | Changes in T0–T1, in T0–T2: Effect, Effect |
|  | Perceived self-efficacy of preconception health promotion [29] | f=0.096 | .166 | No effect |
| *Perception of reproductive health promotion* | |  |  |  |
|  | Self-perception of awareness of preconception health promotion [29] | f=0.413 | <.001 | Effect |
|  | Content awareness of preconception health promotion [29] | f=0.509 | <.001 | Effect |
|  | Perceived benefits of preconception health promotion [29] | f=0.216 | .004 | Effect |
|  | Perceived barriers of preconception health promotion [29] | f=0.231 | <.001 | Effect |
| *Knowledge of reproductive health* | |  |  |  |
|  | Preventive self-management knowledge related to premature labor [30] | f=0.337 | Changes in T0–T1, in T0–T2:  .006 (0.01, 0.11), <.001 (0.09, 027) | Changes in T0–T1, in T0–T2: Effect, Effect |
|  | Genetic knowledge [25] | NR | < .001 | Effect |
| *Perception of hereditary disorders* | |  |  |  |
|  | Perceived severity of mucopolysaccharidosis III [25] |  |  |  |
|  | - Mucopolysaccharidosis III is a severe disease | h=0.277 | < .001 | Effect |
|  | - Mucopolysaccharidosis III has a very bad life expectancy | h=0.224 | < .001 | Effect |
|  | Perceived risk of hereditary disorder [25] |  |  |  |
|  | - Being a carrier of a severe hereditary disease as (very) high risk | h=0.000 | ≥ .05 | No effect |
|  | - Both partners are carrier of the same disease as (very) high risk | h=-0.164 | .001 | Effect |
|  | - Having a child with a severe hereditary disorder as (very) high risk | h=-0.141 | .005 | Effect |
| Reproductive health behaviors | |  |  |  |
| *Self-management for reproductive health* | |  |  |  |
|  | Self-reported discussion of reproductive health with provider [23] | OR^d^=1.97 | .01 (1.22-3.19) | Effect |
|  | Scheduling an additional appointment to address her reproductive health after her well-woman visit [23] | NR | NR | No effect |
|  | Reproductive health promotion behavior [29] | f=0.277 | .001 | Effect |
| *Dietary and nutritional intake* | |  |  |  |
|  | Initiating folate supplementation [23] | NR | NR | No effect |
|  | Folic acid supplement use [28] | Rate ratio=1.02 | (-0.08, 0.26) | No effect |
|  | Dietary risk score [28] | Rate ratio=1.31 | (0.19, 1.34) | Effect |
|  | Vegetable intake [28] | Rate ratio=1.20 | (0.25, 0.86) | Effect |
|  | Fruit intake [28] | Rate ratio=1.00 | (-0.21, 0.39) | No effect |
| *Behavioral promotion on preconception care risks* | |  |  |  |
|  | Preconception care risks from the nutrition domain [22] | |  |  |
|  | - At 6 months, risks that progressed | RR^e^=1.22 | .019 (1.03, 1.45) | Effect |
|  | - At 6 months, risks at action or maintenance | RR=1.26 | .004 (1.08, 1.48) | Effect |
|  | - At 12 months, risks that progressed | RR=1.01 | .928 (0.85, 1.20) | No effect |
|  | - At 12 months, risks at action or maintenance | RR=1.12 | .168 (0.95, 1.31) | No effect |
|  | Reductions in proportion of preconception care risks [27] | h=0.171 | < .01 | Effect |
|  | Reductions in number of preconception care risks per person [27] | h=0.288 | < .05 | Effect |
|  | Preconception care risks [24] |  |  |  |
|  | - At 6 months, risks at action or maintenance | RR=1.16 | <.001 (1.07–1.26) | Effect |
|  | - At 12 months, risks at action or maintenance | RR=1.17 | <.001 (1.08–1.27) | Effect |
|  | - At 6 months, risks that progressed forward | RR=1.17 | .001 (1.06–1.30) | Effect |
|  | - At 6 months, risks that regressed backward | RR=0.83 | .010 (0.72–0.96) | Effect |
|  | - At 12 months, risks that progressed forward | RR=1.07 | .071 (0.97–1.85) | No effect |
|  | - At 12 months, risks that regressed backward | RR=0.84 | .030 (0.72–0.98) | Effect |
| *Contraception use* | |  |  |  |
|  | Initiating or changing the birth control method [23] | OR=0.44 | .03 | Effect |
|  | Initiating reliable contraception use [26] | 6 months: RR=1.45  12 months: RR=1.33 | 6 months: .056 (0.99, 2.12)  12 months: .133 (0.92, 1.91) | 6 months: No effect  12 months: No effect |
|  | Intention to use reliable contraception in the next year [26] | 6 months: RR=1.54  12 months: RR=1.27 | 6 months: .014 (1.03, 2.18)  12 months: .167 (1.09, 1.77) | 6 months: Effect  12 months: Effect |
|  | Initiating dual methods of contraception [26] | Relative risk=1.45 | .052 (1.00, 2.11) | Effect |
|  | Intention to use condoms [26] | 6 months: RR=1.46  12 months: RR=1.43 | 6 months: .027 (1.05, 2.04)  12 months: .035 (1.05, 2.00) | 6 months: Effect  12 months: Effect |

The decimal places of the p-value were basically written up to the 3rd place. However, the p-value with only two decimal places was the value suggested by the original author and could not be calculated in this study.

^a^NR: Not report

^b^f: Cohen's f

^c^h: Cohen's h

^d^OR: odd ratio

^e^RR: rate ratio
